# The Importance of the Detection of Staphylococcus aureus Strain Characteristics Associated With Perioperative Transmission of Antibiotic Resistance

**DOI:** 10.7759/cureus.81885

**Published:** 2025-04-08

**Authors:** Randy W Loftus, Franklin Dexter, Michelle Parra, Soyun M Hwang, Alysha M Robinson, Jonathan E Charnin, Chase P Loftus, Brendan T Wanta

**Affiliations:** 1 Anesthesiology and Perioperative Medicine, Mayo Clinic, Rochester, USA; 2 Anesthesiology, University of Iowa, Iowa City, USA; 3 Anesthesiology, Mayo Clinic, Rochester, USA; 4 Clinical Laboratory, University of Iowa, Iowa City, USA; 5 Research, RDB Bioinformatics, Iowa City, USA

**Keywords:** antibiotic resistance, bacteria, infection control, monitoring, multidrug resistance, multilocus sequence type, pathogenic, perioperative, rapid assay, transmission

## Abstract

Background: Increasing antibiotic resistance among *Staphylococcus aureus* (*S. aureus*) isolates is associated with increased worldwide mortality.Perioperative *S. aureus* transmission is tightly associated with surgical site infection development. Surgical site infections are associated with increased risk of patient mortality, hospital duration, readmission, and increased healthcare expenditure. *S. aureus *multilocus sequence types 5, 8, 105, 30, 59, 72, 188, and 256 are associated with increased risk of the spread of antibiotic resistance between operating rooms on different dates of surgery. In this study, we examined the association of *S. aureus *multilocus sequence types 5, 8, 105, 30, 59, 72, 188, and 256 with 30-day postoperative healthcare-associated infections and postoperative patient cultures obtained for workup of infection.

Methods: Anesthesia workspace reservoirs were previously sampled among 274 case-pairs at three major academic medical centers in the United States. Clonal *S. aureus* transmission was confirmed with whole genome analysis, and patients were followed prospectively for 30-day postoperative healthcare-associated infection development and culture acquisition for workup of infection. The potential association between *S. aureus *multilocus sequence types 5, 8, 105, 30, 59, 72, 188, and 256 detection and clonal transmission with 30-day postoperative healthcare-associated infection and/or patient culture for workup of infection vs. all other anesthesia workspace bacterial isolates was assessed.

Results: Of all anesthesia reservoir isolates, 6% (717/11,664) were associated with healthcare-associated infections and/or postoperative patient cultures. *S. aureus *multilocus sequence types 5, 8, 105, 30, 59, 72, 188, and 256 were associated with greater odds of healthcare-associated infection and/or postoperative culture than other isolates (32% (39/123) of *S. aureus *multilocus sequence types 5, 8, 105, 30, 59, 72, 188, and 256 vs. 6% (678/11,541) other isolates; adjusted odds ratio = 7.45, 95% CI = 3.59-15.46, P < 0.0001). Clonally transmitted *S. aureus *multilocus sequence types 5, 8, 105, 30, 59, 72, 188, and 256 were associated with increased risk of healthcare-associated infection and/or culture vs. other isolates (44% (8/18) *S. aureus *multilocus sequence types 5, 8, 105, 30, 59, 72, 188, and 256 vs. 6% (709/11,646) other isolates; adjusted odds ratio = 14.36, 97.5% CI = 3.14-65.62, P = 0.0002).

Conclusion:* *Detection and clonal transmission of* S. aureus *multilocus sequence types 5, 8, 105, 30, 59, 72, 188, and 256 among anesthesia workspace reservoirs are associated with greater odds of 30-day postoperative healthcare-associated infection development and/or patient cultures vs. other anesthesia workspace isolates.

## Introduction

Increasing antibiotic resistance among *Staphylococcus aureus* (*S. aureus*) pathogens is associated with increased worldwide mortality [1]. The perioperative spread of *S. aureus* is tightly associated with the development of surgical site infections (SSIs) [2,3]. SSIs affect up to 11% of healthy patients undergoing elective surgery [4] and are associated with increased mortality [5], hospital duration [6], hospital readmission [7], and healthcare expenditure [8]. This evidence should provide the impetus for the strategic attenuation of more pathogenic *S. aureus* strain characteristics.

The utilization of *S. aureus* transmission monitoring to optimize perioperative cleaning protocols can achieve substantial reductions in *S. aureus *transmission and all-cause SSIs [3,9]. While practical [10,11] and cost-saving [12], the current monitoring approach is limited by conventional microbiological processing at a central laboratory. Shipping delays and ambient conditions may impact culture yield [13], and sample processing time [13] limits transmission feedback to monthly intervals [3,9,13]. These factors limit real-time attenuation of pathogen transmission events that have the potential to increase the long-term risk of invasive infection development [14,15].

Feedback derived from rapid pathogen detection among monitored reservoirs (e.g., pathogen detection on the anesthesia machine at the start of the first case of a pair indicating a need for improved terminal cleaning of the environment) could be matched with preventive measures during patient care (e.g., ultraviolet-C (UV-C) treatment of the anesthesia machine to prevent subsequent spread of the detected pathogen to the patient) [16]. Additional improvement opportunities include addressing preoperative patient decolonization failures by responding to pathogen detection on patient skin surfaces prior to the surgical incision with repeat decolonization [3,9], implementation of post-discharge decolonization protocols to address patient skin exposures [14,15], or providing practitioner feedback to address hand contamination before or after the incision and/or contamination of the patient intravenous stopcock set [3,9]. While established cleaning protocols [3,9,16] can address these events, biomarkers for heightened antibiotic resistance and pathogenicity are required [3,9].

Recent work has shown that *S. aureus* multilocus sequence types 5, 8, 105, 30, 59, 72, 188, and 256 frequently transmit antibiotic resistance between operating rooms on different days of surgery (longitudinal transmission) [17]. Transmission of antibiotic resistance is particularly problematic because infections are often more difficult to treat, and prolonged antibiotic treatment potentiates the problem by selection of resistance traits [1,2]. For example, *S. aureus* multilocus sequence type 5 is a more pathogenic and antibiotic-resistant strain characteristic [18] with heightened global pathogenicity above that of methicillin-resistant *S. aureus* [19], and it accounts for 63% of longitudinal transmission events involving resistance to the prophylactic antibiotic employed for surgery [17]. Transmission of *S. aureus* multilocus sequence type 5 among anesthesia workspace reservoirs is associated with increased risk of SSI development [20], and pulsed-field gel electrophoresis and whole genome analysis have linked multilocus sequence type 5 transmission to postoperative respiratory infections and SSIs [20,21]. The potential association of *S. aureus* multilocus sequence types 5, 8, 105, 30, 59, 72, 188, and 256 with increased risk of all-cause healthcare-associated infection (HAI) development vs. all other frequently encountered bacterial isolates among anesthesia workspace reservoirs has not been evaluated. This is an important deficit, as the group could serve as a biomarker that could be used by perioperative infection control teams to broadly improve infection control measures and, in parallel, for targeted attenuation of heightened antibiotic resistance and pathogenicity.

Our primary aims for this study were to examine the association of *S. aureus* multilocus sequence types 5, 8, 105, 30, 59, 72, 188, and 256 with all-cause 30-day postoperative HAIs and isolation among postoperative patient cultures obtained for workup of infection. We also sought to characterize their reservoir(s) of origin, and we evaluated the feasibility of the construction of a rapid nucleic acid-based assay for their detection to provide feedback optimization of cleaning protocols [3,9].

## Materials and methods

Background

This analysis involved bacterial pathogens collected from anesthesia reservoirs at three major academic medical centers, including Dartmouth-Hitchcock Medical Center (DHMC) in Lebanon, New Hampshire, the University of Iowa in Iowa City, Iowa, and the UMass Memorial Medical Center in Worcester, Massachusetts, in 2010. A waiver for informed consent was previously obtained (201507774, assessment of routine intraoperative horizontal transmission of potentially pathogenic bacterial organisms) [21]. The University of Iowa determined the use of this de-identified data for this study as non-human subjects’ research without the requirement for patient consent (202404367) [18]. Principal investigators at each site communicated electronically.

We have previously shown stability in the assessment of transmission and prophylactic antibiotic resistance for archival pathogens vs. de novo isolate collection [22]. The isolates used for this study are distinct from those used to identify the role of *S. aureus* multilocus sequence types 5, 8, 105, 30, 59, 72, 188, and 256 strain characteristics in the longitudinal spread of prophylactic antibiotic resistance [17]. The bacterial archive utilized for this analysis was selected because over 11,000 pathogens collected across three major academic medical centers provided the greatest number and variety of pathogens, and therefore, the best platform for assessment of our primary aims [21].

Infection control practices during archival pathogen collection (2010) were like current day practices. All environmental cleaning involved surface disinfection wipes, alcohol dispensers were located on the wall and/or anesthesia carts, and gloves were available [21]. Anesthesia practitioners' hand hygiene was directly observed. Anesthesia practitioners washed their hands < one time per hour at each site, and hand hygiene was not performed following glove removal for 40% of cases [21]. This rate of hand hygiene in 2010 was like that reported in 2015 (0.57 events/hour) among 3,256 operating room environments [23]. Patient enrollment included all-comers to the operating room, and <20% of patients were undergoing orthopedic procedures. As such, few were decolonized preoperatively [21]. Current patient decolonization practices involve only a subset of patients, primarily orthopedic and cardiothoracic [24].

Characterizing the anesthesia workspace isolates

Anesthesia workspace reservoirs were observed in 274 case-pairs involving the first and second case of the day in each operating room. Sampled reservoirs included practitioner hands before, during, and after patient care, the patient nasopharynx and axilla (the groin was not sampled given the focus on anesthesia workspace reservoirs) after induction of anesthesia and patient stabilization, and the anesthesia adjustable pressure-limiting (APL) valve and agent dial of the anesthesia machine before and after the surgical incision. A total of 11,664 bacterial pathogens were isolated and archived for later analysis [21].

The entire surface area of the APL valve and agent dial was actively cleaned with Dimension III before patient entry to the operating room, an approach with proven efficacy [25]. Routine cleaning processes between cases and at the end of the day occurred according to usual standard practice. Bacterial transport swabs (ESwab, Copan Diagnostic Inc., Corona, CA) were used to sample the entire surface of the APL valve and agent dial, patient skin sites, and the patient intravenous stopcock that was primarily used for injection of medications, determined to be so by asking the in-room practitioner(s). Practitioner hands were sampled with a sterile sampling solution (pH 7.9, containing 3.0 g/L NaCl, 0.1 g/L CaCl2, 0.2 g/L KCl, 0.1 g/L MgCl2, 0.2 g/L KH2orally4, and 1.15 g/L K2HPO4) to neutralize residual antiseptic and to disperse bacterial colonies into single cells [21]. All reservoir samples were sent to a central laboratory utilizing overnight shipping to optimize culture yield [13]. Samples were maintained under ambient conditions until initial plating to 5% sheep’s blood agar (SBA) nutrient medium [13,21].

Reservoir isolates were processed by conventional microbiological techniques, including assessment and documentation of bacterial colony morphology, gram stain, simple rapid tests, analytical profile indexing (bioMerieux API identification system, Marcy l'Etoile, France), and antibiotic susceptibility testing [21]. Bacterial susceptibility to ampicillin, cefazolin, cefepime, ceftazidime, cefuroxime, ciprofloxacin, clindamycin, gentamicin, linezolid, meropenem, penicillin, trimethoprim/sulfamethoxazole, tetracycline, methicillin, and vancomycin was recorded and subsequently analyzed as susceptible or resistant [21]. Isolates resistant to ≥ three antibiotics were considered multidrug-resistant (MDR) [26].

DNA was extracted from *S. aureus* isolates and used for next-generation sequencing via the Illumina platform at the Iowa Institute of Human Genetics. Sequence reads were downloaded into the CLC Genomics Workbench Module (version 1.1; Qiagen Aarhus, Germantown, MD) and trimmed to remove adapters and broken pairs. K-mer spectra analysis identified *S. aureus* 252 (MRSA252, NC_002952) as the best reference sequence match for the acquired isolates. All trimmed *S. aureus* sequence reads were mapped to the MRSA252 complete genome, and consensus read maps were analyzed by whole-genome multilocus sequence typing (CLC Genomics Module version 1.1). Genes associated with beta-lactam resistance (e.g., spc, mecA, and blaZ), macrolide resistance (e.g., aadD, ermA, ermC, mphC, inuA, and msrA), aminoglycoside resistance (e.g., aph3III, aac6-aph2, ant(6)-1a), tetracycline resistance (e.g., tetM, and tetK), and fluoroquinolone resistance (e.g., norA) were identified with the microbial genetics module. This approach has been previously tested [18,20].

Clonal transmission

**Figure 1:**
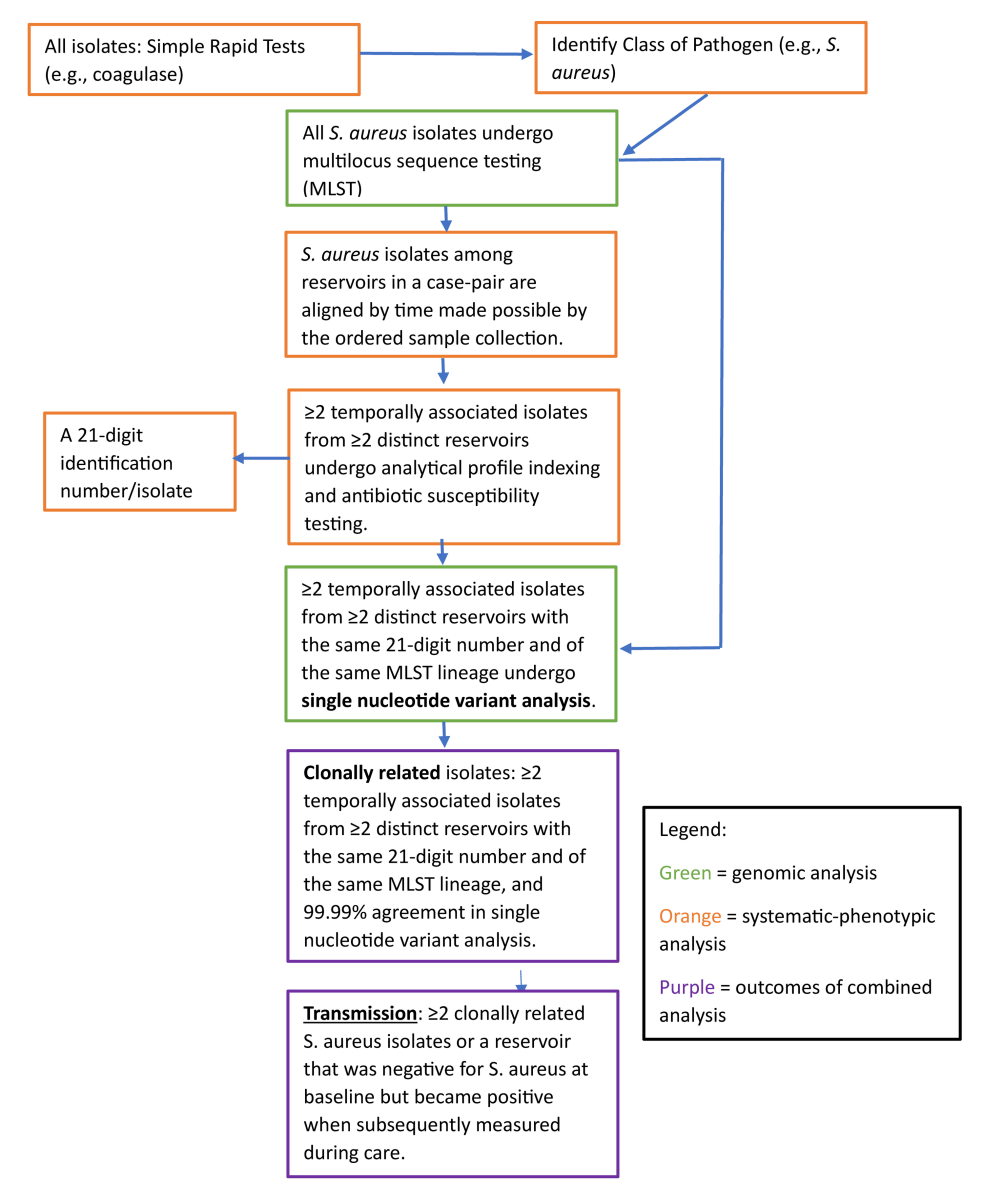
The systematic process used to identify clonal S. aureus transmission. The isolation of ≥2 *S. aureus* isolates from ≥2 distinct anesthesia workspace reservoirs among observed case-pairs that were identical by class of pathogen, analytical profile indexing, antibiotic susceptibility (sensitive, intermediate resistance, or resistance yielding a respective 0, 1, or 2 for each antibiotic and generating a 14-digit number for each isolate for comparison), multilocus sequence type, and with >99.99% agreement in isolate single nucleotide variants (SNVs) among the same multi-locus sequence type [18,20] were considered clonally related.

A systematic-phenotypic-genomic approach was utilized to identify clonal *S. aureus* transmission events (Figure 1).

The isolation of ≥2 bacterial isolates from ≥2 distinct anesthesia workspace reservoirs among observed case-pairs that were identical by class of pathogen, analytical profile indexing, antibiotic susceptibility (sensitive, intermediate resistance, or resistance yielding a respective zero, one, or two for each antibiotic and generating a 14-digit number for each isolate for comparison), multilocus sequence type, and with >99.99% agreement in isolate single nucleotide variants (SNVs) among the same multilocus sequence type [18,20] were considered clonally related.

Transmission origin

The origin of clonally related transmission events was then assessed according to the following approach [18-21].

Practitioner Origin of Transmission

More than or equal to one practitioner hand bacterial isolate(s) was clonally related to ≥ one subsequent, distinct reservoir isolates [18,20,21].

Environmental Origin of Transmission

More than or equal to one bacterial isolate from the anesthesia machine was clonally related to ≥ one subsequent, distinct reservoir isolates [18,20,21].

Patient Origin of Contamination

A bacterial isolate from ≥ one patient's skin surface was clonally related to ≥ one subsequent, distinct intraoperative or postoperative reservoir isolates [18,20,21].

Postoperative healthcare-associated infection and/or patient culture surveillance

Patients and/or patient charts were previously screened for the presence or absence of increased white blood cells, fever, anti-infective order, office visit documenting signs of infection, and/or the acquisition of bacterial cultures for 30 postoperative days during the study period. If positive for ≥ one of these criteria, the principal investigator at each institution confirmed or ruled out 30-day all-cause (wound, respiratory, bloodstream, urinary tract, or other) healthcare-associated infections according to the National Healthcare Safety Network definitions [27,28]. All cultures obtained for workup of infection by clinicians according to standard practice were archived for analysis during the study period. This was accomplished by weekly chart review for each patient and communication with the respective laboratory to save cultures when their acquisition had been documented. Cultures were obtained in the setting of clinical suspicion for infection. Clinical cultures were then available for comparison by the above approach, with surveillance cultures involving anesthesia workspace reservoirs to determine clonal relatedness.

Testing the feasibility for the development of a rapid nucleic acid-based assay

SNVs (CLC Genomics Workbench Plugin, CLC Genomics Module Version 1.1) for commonly encountered and transmitted anesthesia workspace *S. aureus* multilocus sequence types (N = 22) vs. MRSA252 were identified with resequencing analysis. Variant regions were assessed to identify those with 100% capture of all anesthesia reservoir *S. aureus* isolates with either the wild-type or variant allele. Customer-defined amplification primers flanking the variant position were built using the Primer Express 3.0 QuantStudio 6 Flex Real-Time PCR System software (Thermo Fisher Scientific, Waltham, MA). MGB-tagged TaqMan@ probes (DNA Technologies, Coralville, IA) were obtained, and P100 assay performance was assessed via real-time polymerase chain reaction (PCR) across group member (anesthesia workspace reservoir multilocus sequence types) and non-group member archival isolates (other pathogen classes) [29-31].

A total of 30 randomly selected anesthesia reservoir *S. aureus* isolates underwent DNA extraction and dilution to 10 ng/µL. A stock solution of 2.5 µL TaqMan GTXpress Master Mix(2x) (Thermo Fisher Scientific), 0.25 µL of TaqMan genotyping assay mix (20x) (Thermo Fisher Scientific), and 1.25 µL of DNase-free water (Thermo Fisher Scientific) was prepared for 187 reactions (30 *S. aureus* isolates run in triplicate and then duplicated (N = 180) along with six negative controls (N = 6) plus one additional reaction (N = 1)). Four microliters were then pipetted into each of 186 wells of a 384-well plate (Thermo Fisher Scientific) and the plate sealed with an optical adhesive film (Thermo Fisher Scientific) and centrifuged briefly. The film was removed, the diluted DNA sample or water added to each well, and the plate covered with optical adhesive film and centrifuged briefly. The PCR was then executed according to protocol [32].

A total of 175 *S. aureus *isolates, including but not limited to multilocus sequence types 5, 8, 105, 30, 59, 72, 188, and 256, and five other bacterial isolates (*Enterococcus*, *Pseudomonas*, *Acinetobacter*, and *Enterobacter *spp.) underwent DNA extraction and dilution to 10 ng/ µL. A stock solution of 2.5 µL TaqMan GTXpress Master Mix(2x), 0.25 µL of TaqMan genotyping assay mix (20x), and 1.25 µL DNase-free water was prepared for 363 reactions (175 isolates in duplicate (N = 350), five isolates of other bacterial classes in duplicate (N = 10), the negative control with sterile water in duplicate (N = 2), plus an additional reaction (N = 1)). Four microliters were then pipetted into each of 362 wells of a 384-well plate, the plate sealed with an optical adhesive film, and centrifuged briefly. The film was removed, the diluted DNA sample or water (two wells) was added to each well, and the plate was covered with optical adhesive film and centrifuged briefly. The PCR was then executed according to protocol [32].

We quantified *S. aureus* colony-forming units (CFUs) by reservoir of isolation (practitioner hand, patient skin, the anesthesia machine, and the internal lumen of patient intravenous stopcock sets). From this, we learned that clinical environmental samples may yield as little as one CFU, which originates from one bacterium [33], where one *S. aureus* bacterium has a genome of approximately 2.8 million nucleotide base pairs [34], which represents three to five femtograms. Thus, we serially diluted purified DNA from 10 ng/µL to 2.5 fg/µL (a single CFU copy) using 1000-fold dilutions (10 ng/µL, 10 pg/µL, 10 fg/µL, 5 fg/µL, 2.5 fg/µL). A stock solution of TaqMan GTXpress Master Mix(2x), TaqMan genotyping assay mix (20x), and DNase-free water was prepared for 36 reactions, five dilutions for each isolate in triplicate, along with three negative (water) and three positive controls. The solution was then pipetted into each of 36 wells of a 96-well plate (Thermo Fisher Scientific), the plate sealed with an optical adhesive film, and centrifuged briefly. The film was removed, the diluted DNA sample or water added to each well, and the plate covered with optical adhesive film and centrifuged briefly. The PCR was then executed according to protocol [32].

We then evaluated clinical samples of anesthesia reservoirs directly using six samples where *S. aureus* transmission was detected with temporal association and a conventional microbiological approach [13,21] along with six controls involving sterile water. A total of six collection swabs that were previously inserted into one mL of collection buffer (Amies transport medium, ESwab, Copan Diagnostic Inc., Corona, CA) during collection in the clinical environment and following shipment to the central laboratory were vortexed for 15 seconds, and 850 µL were transferred to a separate lysis tube that was centrifuged at 5000 x g (7500 rpm) x 10 minutes. The resultant supernatant was removed, 50 µL lysis buffer (20 mM Tris·Cl, pH 8.0, 2 mM sodium ethylenediaminetetraacetic acid (EDTA), and 1.2% Triton® X-100, Sigma-Aldrich Corp., St. Louis, MO) was added to the lysis tube to resuspend the pellet, and the tube was vortexed for 15 seconds. Lysostaphin (concentration 0.14 mg/mL, Sigma-Aldrich Corp.) was then added to each tube, followed by incubation at 37ºC for 30 minutes and digestion with Proteinase K (final concentration 1.4 mg/mL, Sigma-Aldrich Corp.) for 10 minutes at 65°C [35]. A required assay time of < two hours indicated feasibility for detection and feedback during care, given that most surgical procedures are > two hours in duration [12].

Statistical analysis

All anesthesia workspace isolates were stratified according to healthcare-associated infection and isolation among patient cultures. Fisher’s exact tests were used to compare the proportion of *S. aureus* multilocus sequence types 5, 8, 30, 59, 72, 105, 188, and 256 and all other isolates based on potential covariates. Logistic regression was then used to examine the association of *S. aureus* multilocus sequence types 5, 8, 30, 59, 72, 105, 188, and 256 with all-cause healthcare-associated infection and/or postoperative patient culture acquisition for workup of infection after adjusting for covariates with P < 0.10. The logistic regression was performed using Stata v18.0 (StataCorp, College Station, TX). Robust variance estimates were obtained while clustering by case-pair (i.e., while addressing that errors were probably correlated among isolates of the same case-pair), and P < 0.05 was treated as statistically significant, with 95% two-sided confidence intervals reported for the odds ratios.

In a post hoc analysis, the above was repeated to test the association of *S. aureus* multilocus sequence type 5, 8, 30, 59, 72, 105, 188, and 256 that were clonally transmitted with patient culture obtained for workup of infection alone, where the sample size was insufficient for analysis of healthcare-associated infection alone. P < 0.025 was treated as statistically significant, with 97.5% two-sided confidence intervals reported for the odds and risk ratios, respectively. The logistic regression was then repeated for all *S. aureus* isolates that were clonally transmitted. Because clonally transmitted *S. aureus* isolates were a subset of the multilocus sequence type tested initially, and the same control group was used, Bonferroni-adjustment of the P-value was made for two comparisons, and matching 97.5% confidence intervals were reported for the odds ratio. Simple descriptive statistics were used to compare the proportion of *S. aureus* multilocus sequence types 5, 8, 30, 59, 72, 105, 188, and 256 obtained from the provider hand, patient skin site, and environmental reservoirs.

In subsequent exploratory analyses, this approach was repeated for each pathogen class of all other anesthesia workspace isolates. Because of the multiple comparisons, P-values were reported after Bonferroni-adjustment for 10 comparisons, and 99.5% confidence intervals were reported.

P100 assay performance was assessed by sensitivity, specificity, and utility for the assessment of clinical samples.

## Results

Forty-eight healthcare-associated infections were identified for 44 patients, including urinary tract infections (22/48), superficial and deep surgical site infections (19/48), deep organ space surgical site infections (3/48), and respiratory infections (4/48), with an overall healthcare-associated infection rate of 8% (44/548) [21]. There were 50 postoperative cultures obtained for workup of infection, where 80% (40/50) involved ≥ one pathogen that was epidemiologically related by class of pathogen to ≥ one anesthesia work area reservoir isolate(s). The healthcare-associated infections and patient cultures were from 50 distinct case pairs.

We found that 6% (717/11,664) of all anesthesia workspace isolates were associated with healthcare-associated infections and/or patient cultures (Table 1).

**Table 1:** Anesthesia work area reservoir isolates and all-cause healthcare-associated infections (HAI) and/or isolation among postoperative patient cultures. *Staphylococcus* spp. included the sum of coagulase-negative and beta-hemolytic, coagulase-positive isolates. *S. aureus* multilocus sequence types (MLST) 5, 8, 30, 59, 72, 105, 188, and 256, other *S. aureus* multilocus sequence types, and methicillin-resistant isolates were included in beta-hemolytic and coagulase-positive counts.

	No healthcare-associated infection and/or isolation among postoperative patient cultures (N = 10,947)	Yes healthcare-associated infection and/or isolation among postoperative patient cultures (N = 717)
Organism, N (% of column)		
*Staphylococcus* spp.	5,520 (50.42%)	293 (40.86%)
Coagulase-negative	3,926 (35.86%)	180 (25.10%)
Beta-hemolytic, coagulase-positive	1,594 (14.56%)	113 (15.76%)
Methicillin-resistant	29 (0.26%)	12 (1.67%)
*S. aureus* MLST 5, 8, 30, 59, 72, 105, 188, and 256	84 (0.77%)	39 (5.44%)
Other multilocus sequence types	1,481 (13.53)	62 (8.65)
Gram-negative spp.	2,400 (21.92%)	281 (39.19%)
Fermenting	947 (8.65%)	117 (16.32%)
Nonfermenting	1,453 (13.27)	164 (22.87)
*Enterococcus* spp.	248 (2.27%)	15 (2.09%)
Vancomycin-resistant	14 (0.13%)	3 (0.42%)
Other *Enterococcus* spp.	234 (2.14%)	12 (1.67)
*Streptococcus *spp.	597 (5.87%)	18 (2.51%)
*Corynebacterium* spp.	1,110 (10.14%)	49 (6.83%)
*Micrococcus* spp.	46 (0.42%)	7 (0.98%)
*Bacillus* spp.	1,026 (9.37%)	54 (7.53%)

Among the 274 studied case pairs, there were 272 with complete data for multivariable analysis.

*S. aureus* multilocus sequence types 5, 8, 30, 59, 72, 105, 188, and 256 were associated with greater odds of healthcare-associated infection and/or patient culture than other isolates (32% (39/123) of *S. aureus* multilocus sequence types 5, 8, 30, 59, 72, 105, 188, and 256 vs. 6% (678/11,541) other), with the estimated odds ratio of 7.44. Detection of *S. aureus* multilocus sequence types 5, 8, 30, 59, 72, 105, 188, and 256 stratified by case one, hospital site, male sex, age in years, American Society of Anesthesiologists Health Classification Status (ASA), SENIC index (The National Infections Surveillance (NNIS) and Efficacy of Nosocomial Infection Control (SENIC) Indexes) [36], and patient origin and discharge locations is shown in Table 2.

**Table 2:** S. aureus multilocus sequence types (MLST) 5, 8, 30, 59, 72, 105, 188, and/or 256 detection stratified by all-cause healthcare-associated infection development and/or isolation from postoperative patient cultures. ARMLST: antibiotic-resistant multilocus sequence type; ASA: American Society of Anesthesiologists Health Classification Status; ICU: intensive care unit; SENIC: The National Infections Surveillance (NNIS) and Efficacy of Nosocomial Infection Control (SENIC) Indexes [36]. P-values have been calculated using two-sided Fisher’s exact tests. These do not include the missing values, because the missing values are not used in the multivariable regression. Cramer's V = a statistical measure to assess the strength of association.

	No ARMLST (N = 11,541)	Yes ARMLST type (N = 123)	P-value	Cramér's V
Covariate, N (% of column total)				
Case 1	6,279 (54%)	62 (50%)	0.41	-0.008
Site			0.20	0.018
(1) N=4,854	4812 (42%)	42 (34%)		
(2) N=4,458	4406 (38%)	52 (42%)		
(3) N=2,307	2278 (20%)	29 (23%)		
Missing, N=45	45 (0%)	0 (0%)		
Male (N=5,169)	5101 (44%)	68 (55%)	0.022	0.024
Missing, N=118	118 (1%)	0 (0%)		
Age			0.028	0.024
18 to 64 years, N=8642	8548 (74%)	94 (76%)		
65 to 79 years, N=2447	2418 (21%)	29 (24%)		
80+ years, N=461	461 (4%)	0 (0%)		
Missing, N=114	114 (1%)	0 (0%)		
ASA				
1-2 (N=1133)	1148 (10%)	11 (9%)	0.41	0.023
3 (N=7039)	6962 (60%)	77 (63%)		
4 (N=3093)	3063 (27%)	30 (24%)		
5 (N=203)	198(2%)	5 (4%)		
Missing (N=170)	170 (1%)	0 (0%)		
SENIC			0.015	0.034
0 (N=4005)	3945 (34%)	60 (49%)		
1 (N=5136)	5090 (44%)	46 (37%)		
2 (N=1996)	1980 (17%)	16 (13%)		
3 (N=400)	439 (4%)	1 (1%)		
4 (N=42)	42 (0%)	0 (0)		
Missing (N=45)	45 (0%)			
Patient origin location			0.026	0.030
Same day (N=10,613)	10,508 (91%)	105 (85%)		
Hospital floor (N=738)	725 (6%)	13 (11%)		
ICU (N=177)	172 (1%)	5 (4%)		
Other (N=90)	90 (1%)	0 (0%)		
Missing (N=46)	46 (0%)	0 (0%)		
Patient discharge location			0.0074	0.034
Same day (N=5352)	5288 (46%)	64 (52%)		
Hospital floor (N=5589)	5541 (48%)	48 (39%)		
ICU (N=464)	453 (4%)	11 (9%)		
Other (N=214)	214 (2%)	0 (0%)		
Missing (N=45)				

Patient sex, age, SENIC index, origin location, and discharge location had univariable P < 0.10. There was a significant increase in the odds of association with healthcare-associated infection and/or patient culture for *S. aureus *multilocus sequence types 5, 8, 30, 59, 72, 105, 188, and 256 vs. all other anesthesia work area isolates following adjustment for these potential covariates (adjusted odds ratio = 7.45, 95% CI = 3.59-15.46, P < 0.0001) (Table 3).

**Table 3:** Logistic regression analysis of the association of detection of S. aureus multilocus sequence types 5, 8, 30, 59, 72, 105, 188, and 256 with all-cause 30-day healthcare-associated infections and/or isolation among postoperative patient cultures. The logistic regression was estimated using robust variance estimation clustered among the 272 case pairs and 11,524 isolates with complete data. Covariates are listed in the sequence of Table 2. SENIC = an index predicting the probability of postoperative healthcare-associated infection development for a given patient based on general abdominal surgery, case duration of > two hours, > two comorbidities, and dirty or infected site [36].

Covariate	Odds ratio	95% confidence interval	P-value
*S. aureus* multilocus sequence types 5, 8, 30, 59, 72, 105, 188, and 256	7.45	3.59 to 15.46	<0.0001
Female	1.26	0.71 to 2.22	0.42
Age, baseline 18 to 64 years	-	-	-
65 to 79 years	1.52	0.79 to 2.91	0.21
80+ years	1.03	0.26 to 4.03	0.97
SENIC, baseline 0	-	-	-
1	0.85	0.36 to 2.00	0.72
2	1.07	0.46 to 2.50	0.87
3	1.35	0.38 to 4.84	0.64
4	8.90	1.07 to 73.85	0.043
Patient origin location, baseline same day	-	-	-
Floor	1.16	0.45 to 2.97	0.77
ICU	2.03	0.36 to 11.39	0.42
Discharge location	-	-	-
Floor	1.12	0.53 to 2.37	0.77
ICU	2.01	0.40 to 10.19	0.40
Other	1.08	0.13 to 8.88	0.94

The confidence interval was appropriately wide based on healthcare-associated infections and/or patient cultures being from 50 distinct case pairs; see first paragraph. The Cornfield confidence interval for odds ratio was much narrower (5.06-10.94), and the logistic regression confidence interval using regular standard errors was comparable (5.01-11.09). *S. aureus* multilocus sequence types 5, 8, 30, 59, 72, 105, 188, and 256 were also associated with increased risk of patient culture alone vs. all other anesthesia work area isolates (32% (39/123) *S. aureus* multilocus sequence types 5, 8, 30, 59, 72, 105, 188, and 256 vs. 6% (675/11,541) other; adjusted odds ratio = 7.48, 97.5% CI = 3.25-17.23, P < 0.0001).

Clonally transmitted *S. aureus* multilocus sequence types 5, 8, 30, 59, 72, 105, 188, and 256 were associated with increased risk of healthcare-associated infection and/or patient culture vs. other isolates (44% (8/18) *S. aureus* multilocus sequence types 5, 8, 30, 59, 72, 105, 188, and 256 vs. 6% (678/11,541) other; adjusted odds ratio = 14.36, 97.5% CI = 3.14-65.62, P = 0.0002). There was also a significant association of clonally transmitted *S. aureus *multilocus sequence types 5, 8, 30, 59, 72, 105, 188, and 256 with patient culture alone (44% (8/18) *S. aureus* multilocus sequence types 5, 8, 30, 59, 72, 105, 188, and 256 vs. 6% (706/11,646) other; adjusted risk ratio = 14.40, 97.5% CI = 3.15-65.8, P < 0.0001). Clonal transmission stories are summarized in Table 4.

**Table 4:** Transmission stories for S. aureus among anesthesia workspace reservoirs. CRNA: certified registered nurse anesthetist.

Multilocus sequence type	Origin	Transmission location	Prophylactic antibiotic	Resistance prophylactic antibiotic	mecA
5	Unknown	Resident physician hand end case 1	Clindamycin	Yes	Yes
5	Unknown	Anesthesia machine end case 2	Cefazolin	Yes	Yes
5	Resident hand start case 1	Patient axilla and nose, machine	None	N/A	Yes
		Case end			
5	Unknown	CRNA hand end case 2	None	N/A	Yes
5	Patient axilla and nose	Patient infection	Cefazolin/clindamycin	Yes	Yes
5	Patient nose case 2	Infection	Cefazolin	Yes	Yes
5	Patient nose case 1	Other provider hand during care	Vancomycin/cefazolin	No	No
5	Unknown	CRNA hand beginning/end case 2	None	N/A	No
8	Anesthesia machine end case 2	Patient infection	Cefazolin	Yes	Yes
8	Other provider during care	Attending hand start case 1	Cefazolin	Yes	Yes
8	Other provider during care	Attending hand start case 2	Vancomycin/cefuroxime	No	Yes
8	Patient axilla case 1	Patient axilla case 2, stopcock case 2,	Cefazolin	No	No
		Anesthesia machine end case 2,			
		Other provider hand during care			
8	Patient nose case 2	Other provider hand during care	Cefazolin	No	No
8	Patient nose case 2	Infection	None	N/A	Yes
15	Unknown	Attending physician hand start case 1 and 2	Gentamicin/cefazolin	No	No
15	Attending hand start case 1	Other provider hand during care	Cefazolin	No	No
15	Unknown	Attending hand end case 2	None	N/A	No
30	Patient nose case 1	Patient axilla case 2	Vancomycin/cefazolin	No	No
30	Patient nose case 2	Anesthesia machine end case 2	Ampicillin-sulbactam	No	No
30	Patient nose case 1	Patient nose case 2	Cefazolin/clindamycin	No	No
30	CRNA hand start case 1	Other provider hand during care	Cefazolin/clindamycin	No	No
59	Patient nose start case 2	CRNA hand end case 2, other provider	Cefazolin/clindamycin	No	No
		Hand during care			
59	Patient nose start case 2	CRNA hand beginning and end case 2	Cefazolin/clindamycin	No	No
188	Patient nose start case 2	Infection	Cefazolin/clindamycin	No	Yes
1049	Resident hand start case 1	Patient nose start case 1	Vancomycin	No	No

The summary includes the reservoir of origin, transmission location(s), prophylactic antibiotic administered, resistance (yes/no) to the administered antibiotic, presence of the mecA trait, and MDR. Confirmed reservoirs of origin for *S. aureus* multilocus sequence type 5, 8, 30, 59, 72, 105, 188, and 256 included provider hands (45%, 55/123), patient skin sites (41%, 50/123), and the anesthesia machine (10%, 12/123). Approximately 70% (5/7) of transmission stories involving MDR were *S. aureus* multilocus sequence type 5. *S. aureus* multilocus sequence types 5, 8, 30, 59, 72, 105, 188, and 256 accounted for approximately 84% (21/25) of confirmed transmission events.

When repeated for all other classes of anesthesia workspace isolates, coagulase-positive *Staphylococcus* (OR = 3.05, 99.5% CI = 1.37-6.78, adjusted P = 0.0009), methicillin-resistant *S. aureus* (OR = 6.07, 99.5% CI = 1.47-25.15, adjusted P = 0.0037), gram-negative spp. (OR = 2.32, 99.5% CI = 1.73-3.12, adjusted P < 0.0001), and fermenting gram-negative spp. (OR = 2.11, 99.5% CI = 1.52-2.94, adjusted P < 0.0001) were associated with increased odds of association with healthcare-associated infection and/or patient culture, while *Streptococcus spp.* (OR = 0.45, 99.5% CI = 0.21-0.94, adjusted P = 0.025) and coagulase-negative *Staphylococcus* (OR = 0.60, 99.5% CI = 0.50-0.71, adjusted P < 0.0001) were associated with reduced odds of association with healthcare-associated infection and/or patient culture. A lower proportion of *S. aureus* multilocus sequence types 5, 8, 30, 59, 72, 105, 188, and 256 was associated with anesthesia reservoir isolation of coagulase-negative* Staphylococcus* (22.76% (28/123) vs. 35.33% (4,078/11,541) other reservoir isolates, P = 0.004).

**Figure 2:**
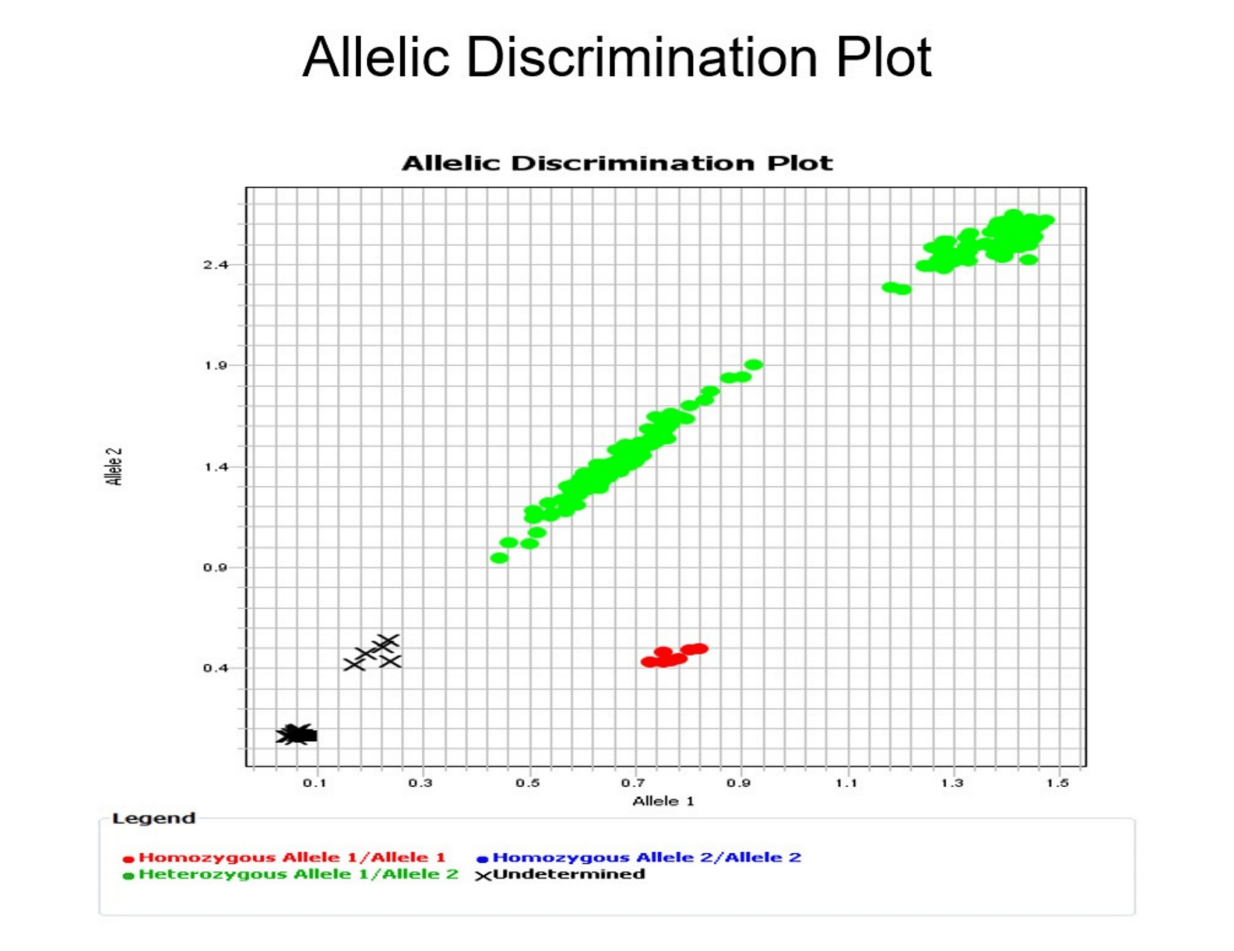
P100 rapid nucleic-assay. P100 assay (wild-type allele MRSA252 (red dots) and other strains (green dots) for *S. aureus* multilocus sequence types that are frequently transmitted among anesthesia workspace reservoirs [17,18,20], including multilocus types 5, 8, 105, 30, 59, 72, 188, and 256 (green and red dots) vs. negative controls (bottom left corner) and other pathogens (x).

The P100 assay had observed 100% sensitivity and specificity for detection of 22 *S. aureus* sequence types, including but not limited to *S. aureus* multilocus sequence types 5, 8, 30, 59, 72, 105, 188, and 256, commonly isolated among anesthesia workspace reservoirs and implicated in transmission vs. non-*S. aureus* isolates (Figure 2).

We were able to amplify to 2.5 femtograms, one copy of the *S. aureus* genome, and the capacity for direct detection from clinical samples was confirmed, where 1/6 samples (patient groin) that were evaluated positive for *S. aureus* by conventional microbiological techniques amplified with the P100 assay, along with the positive control. Point-of-care interventions that could be guided by rapid feedback regarding rapid detection of these strain characteristics are summarized in Table 5.

**Table 5:** S. aureus multilocus sequence transmission events matched and proposed interventions during patient care driven by P100 rapid assay detection.

Multilocus sequence type 5
Resident physician hand contamination during care, direct feedback of contamination and reinforcing importance of washing hands to prevent colonization with multidrug resistance (MDR) strain characteristics, clean the environments you have interacted with now using surface disinfection wipes [3,9], if repeated exposure and confirmation of viability, decolonization protocol, treat exposed environment with triangular ultraviolet-C (UV-C) irradiation for two minutes before the next case [16].
Limitation of conventional: Processing time would not allow timely individual feedback to wash their hands or to clean the environment they interacted with [13].
Anesthesia machine contamination end of patient care, feedback to involved anesthesia practitioners during care that a post-induction wipe down of the machine/environment [3,9] is important to prevent spread of multidrug resistance (MDR), that noncompliance resulted in machine contamination that can subsequently spread longitudinally and impact other patients and providers, and as such, treat the anesthesia work area for two minutes with UV-C using a triangular configuration to augment surface disinfection cleaning before the next case [16].
Limitation of conventional: Processing time would not allow timely feedback for targeted environmental cleaning [13].
Resident hand contaminated at the start of case 1, direct feedback to the individual that noncompliance in hand hygiene resulted in patient and machine contamination with a MDR pathogen, you have been exposed, wash your hands now, if repeated detection, consider decolonization, for the exposed patient (nose and axilla), implement post-discharge decolonization protocol [14,15], for the machine, two minutes of UV-C treatment using a triangular configuration [16].
Limitation of conventional: Processing time would not allow timely feedback to the individual to address their hand contamination and for targeted environmental cleaning, or to decolonize the exposed patient prior to discharge [13]. This would not allow the prevention of the spread of resistance from the hospital to the community.
Certified registered nurse anesthetist (CRNA) hand contamination end case 2, direct feedback to the individual, you have been exposed, wash your hands now, if repeated detection, consider decolonization if confirm viability.
Limitation to conventional: Processing time would not allow timely individual feedback to the exposed provider to wash their hands now to prevent secondary transmission events [13].
Patient axilla and nose contamination at the start of case 1 and infection development, direct feedback to the surgeon regarding the importance of patient decolonization [3], assess the decolonization process, and implement post-discharge patient decolonization [14,15].
Limitation to conventional: Processing time would not allow timely feedback to the patient to implement decolonization before discharge to help prevent the development of downstream infection by protecting the wound from exposure during epithelialization [13].
Multilocus sequence type 8
Contamination of the anesthesia machine at the end of case 2, feedback to anesthesia practitioners involved regarding the importance of the post-induction wipe down in preventing spread of MDR [3,9], targeted cleaning of the machine with two minutes of UV-C using a triangular configurationbefore the next case [16], communicate the impact on patient infection, and for the patient, implement post-discharge decolonization [14.15]
Limitation to conventional: Processing time would not allow timely, targeted cleaning of the exposed machine and addressing patient decolonization prior to discharge to prevent the development of the infection [13].
Attending hand contamination at the start of the case affecting another provider, direct feedback to communicate the importance of hand hygiene before and during patient care to prevent the spread of MDR, how one provider can impact another, wash your hands now, if detected again, consider decolonization.
Limitation to conventional: Processing time would not allow timely feedback to the attending provider that they started a case contaminated with a MDR pathogen that spread to another provider due to noncompliance, wash hands now, start decolonization protocol, to affected provider, wash hands now, if detect again, consider decolonization protocol [13].
In all cases, continue monitoring and proactive improvement.

## Discussion

Increasing antibiotic resistance is associated with increased worldwide mortality, with resistance among *S. aureus* isolates being a leading cause [1]. Perioperative *S. aureus* transmission is associated with increased risk of surgical site infection development. *S. aureus* multilocus sequence types 5, 8, 105, 30, 59, 72, 188, and 256 are frequently encountered among anesthesia workspace reservoirs and are associated with increased risk of perioperative spread of resistance between operating rooms on different days of surgery (longitudinal spread) [17]. In this study, we show that these strain characteristics are also associated with increased risk of 30-day postoperative healthcare-associated infection development and/or patient cultures obtained for workup of infection [3,9].

Improved perioperative cleaning procedures can generate substantial reductions in *S. aureus* transmission and all-cause surgical site infections [3,9]. *S. aureus* transmission monitoring among anesthesia workspace reservoirs can be used to optimize cleaning protocols [10-12]. The current approach to monitoring is limited by conventional microbiological processing at a central laboratory that may limit culture yield [13] and by a sample processing time that limits feedback to monthly intervals [3,9]. These factors prohibit the attenuation of pathogen transmission at the patient level during their perioperative care period. As such, unmitigated pathogen transmission events to patients during their care can increase the risk of all-cause healthcare-associated infections for up to one year following the exposure [14,15]. An example of such a transmission event includes transmission from the anesthesia machine, the most potent intraoperative transmission vehicle [21], to patient skin surfaces during surgery. Such transmission events are proven to be tightly associated with surgical site infection development [2] and often involve antibiotic-resistant strain characteristics [22], the latter diverging from pathogens isolated from the surgical incision that are invariably sensitive to the prophylactic antibiotic employed [37]. SSIs, however, are not the only cause of perioperative patient harm derived from infection. Patients also suffer from primary and secondary bloodstream infections, healthcare-associated pneumonia, and urinary tract infections [21,38,39]. An ideal approach for perioperative infection prevention would therefore impact all-cause healthcare-associated infections.

The development of a perioperative point-of-care infection prevention program to attenuate the spread of bacterial pathogens with heightened antibiotic resistance and pathogenicity to reduce all-cause healthcare-associated infections is the next logical step. However, programmatic development requires knowledge of biomarkers for heightened antibiotic resistance and pathogenicity that can be targeted for prevention. Recent work has shown that *S. aureus* multilocus sequence types 5, 8, 105, 30, 59, 72, 188, and 256 are associated with increased risk of longitudinal spread of antibiotic resistance [17] while Gram-negative and *Enterococcus* spp. are not [40]. In another study, Gram-negative organisms were rarely isolated from anesthesia workspace environmental reservoirs, and *Enterococcus* organisms were rarely isolated from intravenous stopcock sets [41]. Thus, Gram-negative and *Enterococcus* spp. are unsuitable for monitoring to provide feedback for optimization of cleaning protocols in the anesthesia workspace [41]. Taken together [2,17,40,41], *S. aureus* is the sentinel pathogen for monitoring to provide feedback for optimization of cleaning protocols. In this study, we extend this body of knowledge by examining the potential association of *S. aureus* multilocus sequence types 5, 8, 105, 30, 59, 72, 188, and 256 with increased risk of all-cause 30-day healthcare-associated infections and/or postoperative patient culture development.

As compared to all other anesthesia workspace isolates, we found that *S. aureus* multilocus sequence types 5, 8, 105, 30, 59, 72, 188, and 256 are associated with greater odds of all-cause healthcare-associated infections and/or postoperative patient cultures following appropriate adjustment for potentially confounding variables. Clonal transmission of these strain characteristics was associated with even greater odds of healthcare-associated infection and/or patient culture, consistent with prior work showing that transmission of antibiotic-resistant isolates among anesthesia workspace reservoirs is associated with increased risk of infection development [2,22] and adding to prior work by the extension from surgical site infections to all-cause healthcare-associated infections. The validity of these results is supported by the expected association of other major bacterial pathogens (e.g., Gram-negative spp.) with increased risk of healthcare-associated infections and/or patient culture and the association of *coagulase-negative Staphylococcus* and *Streptococcus* spp. with reduced odds of healthcare-associated infection and/or patient culture [42]. While *S. aureus *and Gram-negative pathogens have an increased risk of harboring virulence and antibiotic-resistant traits, coagulase-negative* Staphylococcus* and *Streptococcus spp*. are not as likely to do so [43]. *S. aureus* is among a set of pathogens that are likely to be more antibiotic resistant and more virulent (vancomycin-resistant *Enterococcus*, *S. aureus*, *Klebsiella pneumoniae*, *Acinetobacter baumannii*, *Pseudomonas aeruginosa*, and *Enterobacter* spp., ESKAPE) [43], so the confirmed association of *S. aureus* with increased risk of healthcare-associated infection and/or patient cultures obtained for workup of infection vs. all other anesthesia workspace bacterial pathogens was an expected finding that required confirmation. These results, in the context of what is known about the associated increased risk of antibiotic resistance, thereby provide evidence that supports the incorporation of *S. aureus* multilocus sequence types 5, 8, 105, 30, 59, 72, 188, and 256 detection into a point-of-care perioperative infection prevention program. This may help to address the worldwide problem of increasing antibiotic resistance associated with increased mortality [1]. This potential impact would not be solely due to intervention in the perioperative arena but by extension of what is learned from the perioperative arena to other healthcare environments (e.g., postoperative and outpatient settings, nursing homes, ambulatory care settings) and to emerging pathogens and associated strain characteristics. To achieve the greatest impact, future work should strive to determine, using the genomic approach outlined in this study, the relative contribution of pathogen spread in different settings to the development of infection and/or positive patient cultures.

In anticipation of these findings, we considered a priori the analysis of *S. aureus *multilocus sequence types 5, 8, 105, 30, 59, 72, 188, and 256 transmission dynamics. We found that confirmed reservoirs of origin included practitioner hands, patient skin sites, and the anesthesia machine. Multilocus sequence types 5, 8, 105, 30, 59, 72, 188, and 256 together explained 84% of MDR clonal *S. aureus* transmission events. These findings are consistent with prior work [17,20,21], further strengthening the validity of these study results.

We report the feasibility of the development of a rapid assay for the detection of these strain characteristics. The P100 assay has 100% sensitivity for 22 commonly encountered *S. aureus* multilocus sequence types among anesthesia workspace reservoirs, including but not limited to multilocus sequence types 5, 8, 105, 30, 59, 72, 188, and 256 [17,18,20,22]. It is also specific for *S. aureus* vs. other anesthesia workspace pathogens. Thus, the assay could be used to broadly improve infection control protocols while targeting the attenuation of these strain characteristics that are now with confirmed heightened antibiotic resistance [17] and pathogenicity. We report a reasonable limit of detection, including for potentially low-yield environmental surfaces, and we have confirmed direct detection from swab transport systems. Thus, use of the rapid assay that we have developed could be of wide utility, including for epidemiological field studies [13].

A next step, however, is to use the P100 assay to provide feedback to improve infection control measures during perioperative patient care. *S. aureus* multilocus sequence types 5, 8, 105, 30, 59, 72, 188, and 256 transmission events are summarized in Table 4, and additional benefits potentially derived from rapid P100 assay detection are in Table 5. We utilized actual transmission events (Table 4) to explain the potential benefit derived from assay use (Table 5) when P100 detection is matched with therapeutic interventions during patient care. For example, anesthesia machine P100 positivity could be addressed with two minutes of triangular UV-C treatment proven effective against the more pathogenic *S. aureus *multilocus sequence type 5 [16] that has increased strength of biofilm formation and desiccation tolerance [18]. Another example would be the use of practitioner hand P100 detection to provide direct feedback shown to be an important component of hand hygiene compliance for anesthesia practitioners [9], a highly innovative approach for hand hygiene augmentation, or likewise, to provide direct practitioner feedback to improve vascular care via leveraging an approach that has also been proven to be effective against the more pathogenic *S. aureus *multilocus sequence type 5 (transmission to stopcock set with multilocus sequence type 8 confirmed and shown in Table 4) [44]. Finally, the P100 assay could be used to provide feedback to guide implementation of post-discharge patient decolonization protocols proven to reduce all-cause healthcare-associated infections [14,15]. The latter is particularly important, as five days of nasal and skin decolonization for each of five consecutive months for patients with confirmed healthcare pathogen exposure is proven to generate a substantial reduction in the risk of invasive methicillin-resistant *S. aureus* and all-cause healthcare-associated infections vs. patient education alone [14,15]. However, this proven protocol has not achieved widespread implementation in the healthcare setting. Thus, a point-of-care approach leveraging the P100 assay is likely to generate substantial reductions in all-cause 30-day healthcare-associated infections when matched to proven therapeutic measures [9,14-16,44] simply by improving implementation compliance. Future work, however, should evaluate the logistics of P100 detection, feedback [3,9], and therapeutic implementation [14-16,44], followed by formal analysis of the impact of the approach on all-cause 30-day postoperative healthcare-associated infections.

Limitations and strengths

We utilized archival isolates. We have confirmed that the isolates used for the analysis [21] have stable strain characteristics vs. isolates collected de novo [22]. Thus, the acquisition of de novo isolates for the purpose of this analysis, while possible, would have required substantial financial resources without a clear scientific rationale. We further support this premise by explaining the consistency in infection control practices at the time of the archival collection vs. current practices [17-24], and we have shown consistency in the reported data regarding expected and previously reported findings. We utilized the combined analysis of healthcare-associated infection and patient culture, an evidence-based approach [45]. This approach leverages clinical chart review along with surveillance and clinical cultures to accurately document infections [45]. Infections were reviewed within 30 days of the procedure [21], and patient charts were reviewed weekly to facilitate communication with the clinical laboratories to save cultures obtained for workup of infection [21]. We carefully modeled the impact of potentially confounding covariates such as SENIC indices [36]. Future work could extend this approach to the 90-day postoperative period [46] to capture a greater incidence of healthcare-associated infections and/or patient cultures. This could be achieved in parallel with analysis of the impact of the point-of-care program with rapid P100 detection, feedback, and treatment on clinical patient cultures, entirely appropriate when analyzing transmission and/or the impact of a treatment approach. The importance of transmission feedback is clearly established for both a multifaceted perioperative infection control approach and for the individual components [47].

## Conclusions

The results of this study show that *S. aureus* multilocus sequence types 5, 8, 105, 30, 59, 72, 188, and 256 are associated with increased risk of healthcare-associated infection and/or patient culture vs. all other anesthesia workspace pathogens. This heightened pathogenicity vs. all other anesthesia workspace isolates, along with the previously reported increased risk of longitudinal spread of antibiotic resistance, supports their incorporation into an evidence-based *S. aureus* transmission monitoring protocol to further enhance cleaning protocol optimization for 30-day postoperative healthcare-associated infection prevention. Further work is indicated to evaluate the efficacy of this approach for reducing 30-day postoperative healthcare-associated infections.
